# The Treatment of Fractured Patella

**Published:** 1900-02-10

**Authors:** 


### THE TREATMENT OF FRACTURED PATELLA.

For suturing the patella after recent fracture Mr. Glasgow Patteson,[1] of the Meath County Hospital, recommends the use of a broad silver wire having a section of a D-shape, flat on one side and round on the other. Among the most important points connected with the operation he emphasises the following: (1) Delay in operating for a few days after the injury; (2) thorough clearing out of the joint, all clots being removed and the broken surfaces thoroughly cleaned; (3) the use of an easily sterilisable suture of silver wire as described, so shaped as to render splitting of the bone an impossibility; (4) the elevation and not the resection of the fibrous curtain, and its subsequent accurate suturing over the embedded wire; (5) the early commencement of passive movement.

[1] Brit. Med. Jour., Feb. 3.

